# Effects of Different Solvents Extractions on Total Polyphenol Content, HPLC Analysis, Antioxidant Capacity, and Antimicrobial Properties of Peppers (Red, Yellow, and Green (*Capsicum annum* L.))

**DOI:** 10.1155/2022/7372101

**Published:** 2022-01-19

**Authors:** Ahmad Mohammad Salamatullah, Khizar Hayat, Fohad Mabood Husain, Mohammed Asif Ahmed, Shaista Arzoo, Mohammed Musaad Althbiti, Abdulhakeem Alzahrani, Bandar Ali M. Al-Zaied, Heba Kahlil Alyahya, Nawal Albader, Hiba-Allah Nafidi, Mohammed Bourhia

**Affiliations:** ^1^Department of Food Science & Nutrition, College of Food and Agricultural Sciences, King Saud University, P. O. Box 2460, Riyadh 11451, Saudi Arabia; ^2^Department of Food Science, Faculty of Agricultural and Food Sciences, University Street, 2325 Quebec City, QC, Canada; ^3^Laboratory of Chemistry Biochemistry, Environment, Nutrition and Health, Faculty of Medicine and Pharmacy, Hassan II University, Casablanca 5696, Morocco

## Abstract

Plants possessing various bioactive compounds and antioxidant components have gained enormous attention because of their efficacy in enhancing human health and nutrition. Peppers (*Capsicum annuum* L.), because of their color, flavor, and nutritional value, are considered as one of the most popular vegetables around the world. In the present investigation, the effect of different solvents extractions (methanol, ethanol, and water) and oven drying on the antioxidant and antimicrobial properties was studied of red, yellow, and green peppers. The green pepper water extract showed the highest total polyphenol content (30.15 mg GAE/g DW) followed by red pepper water extract (28.73 mg GAE/g DW) and yellow pepper water extract (27.68 mg GAE/g DW), respectively. The methanol extracts of all the pepper samples showed higher TPC as compared to the ethanol extract. A similar trend was observed with the total flavonoid content (TFC). The antioxidant assays (DPPH scavenging and reducing power) echoed the findings of TPC and TFC. In both antioxidant assays, the highest antioxidant activity was shown by the water extract of green pepper, which was followed by the water extract of red pepper and yellow pepper. Furthermore, all extracts were assessed for their potential antimicrobial activity against bacterial and fungal pathogens. Aqueous extracts of all three pepper samples exhibited slightly higher inhibition zones as compared to their corresponding ethanolic and methanolic extract. Minimum inhibitory concentration (MIC) values ranged from 0.5 to 8.0 mg/ml. The lowest MIC values ranging from 0.5 to 2.0 mg/ml concentration were recorded for aqueous extracts of green pepper. High-performance liquid chromatography (HPLC) analysis revealed tannic acid as the major phenolic compound in all three pepper samples. Thus, it is envisaged that the microwave drying/heating technique can improve the antioxidant and antimicrobial activity of the pepper.

## 1. Introduction

Antioxidants protect biological processes by delaying, controlling, or inhibiting the oxidative stress caused by free radicals [1]. Free radicals' accumulation in the human body could disturb the normal functions of cells and organs that successively result in the onset of noncommunicable diseases (NCDs) [2]. Plants with a variety of bioactive compounds and antioxidant components are gaining popularity as a result of their efficacy in enhancing human health and nutrition [3, 4]. They have been linked to lower cancer and heart disease incidence and in turn the mortality rates [5, 6].

Peppers (*Capsicum annuum* L.) are a member of the Solanaceae family known by various other names too, such as bell pepper, chili, and capsicum. Because of their color, flavor, and nutritional value, peppers are considered one of the most popular vegetables around the world. The plant, which is native to North and South America, thrives in hot, dry climates and is used in Africa and other parts of the world for both medicinal and culinary purposes [7]. They are thick-walled bell-shaped vegetables, comprising three or four lobes, and are found in different sizes and colors depending on the genotype or seasonal period of breeding [8]. The chlorophyll and carotenoids give peppers their green color [9, 10], and carotene, zeaxanthin, lutein, and cryptoxanthin are liable for giving the yellow-orange hue of pepper [10]. Capsanthin, capsorubin, and capsanthin 5,6-epoxides are carotenoid pigments that give peppers their red color [11]. The difference in levels of these compounds, changes during ripening, the genotype, and the seasonal period of breeding are the various factors responsible for the differences in the colors of peppers. The taste and flavor of each pepper can be influenced by the color of the fruit. Red, yellow, and orange peppers, for example, are sweeter than green peppers as a result of higher glucose content during the ripening period [12]. Bell peppers are good sources of vitamins, such as vitamins C and E, provitamin A, and carotenoids [13–15]. They were also found to be a good source of phenolic or flavonoids, such as quercetin, luteolin, and capsaicinoids [16]. Types and quantities of bioactive compounds differ among different colored peppers.

Studies have shown the efficacy of the antioxidative components of several pepper species [17, 18]. They are effective in reducing the risk of various degenerative, mutagenic, and chronic diseases [19–21]. It has also been used for alleviating toothaches and in the management of the respiratory disease [22]. Loizzo et al. 2008 reported the inhibitory effect of *C. annuum* var. Acuminatum on the enzyme acetylcholinesterase, which is a therapeutic method for the symptomatic management of Alzheimer's disease [23]. In animal assays, peppers have shown hypocholesterolemic properties [24, 25]. Capsaicin, the main representative of the pungent components, is a lipophilic alkaloid and because of its analgesic and anti-inflammatory activity has been used in clinical practice. An analysis on rats' revealed peppers antioxidant capacity, which has defensive effects on the brain cells [26]. It is critical to study the phytochemicals found in common vegetables and fruits in order to learn more about their possible health benefits. The extraction solvents used may have an impact on the precision with which bioactive compound concentrations are measured [27, 28]. In natural foods, the concentration and activity of bioactive compounds can be directly related to solvent properties such as lipophilic and hydrophilic solvents and their respective polarity [14, 29]. A study on the efficiency of different extraction solvents (hexane, ethyl acetate, acetone, methanol, and methanol-water mixture) using high-performance liquid chromatography (HPLC) on the bioactivity of nonpungent peppers demonstrated that solvent chemical properties such as polarity can differentially influence the efficacy of recovering bioactive compounds from foods, and this might eventually result in differences in estimated biological activity, such as antioxidant capacity [30]. In another study, in comparison with green and yellow sweet peppers, the orange and red sweet peppers extracted with hexane showed the highest TPCs and antioxidant activities, likely caused by carotenoids as the compounds were mainly extracted by nonpolar solvents [31]. Perishable products face losses due to enzymatic and microbial degradation which are active at suitable temperature and storage problems. Drying comprises concurrent transient heat, mass, and momentum transport, and it is one of the most widely used methods for the preservation of food [32]. Dried food products can be stored at ambient temperatures for longer periods due to their low moisture content, which reduces the microbiological activity and allows the availability of the product even in off-season. Studies have indicated that as substitutes to the conventional drying procedures (sun drying), the use of microwaves drying/heating techniques can improve the antioxidant activity of the plant materials by reducing the thermal damages of antioxidant components [33, 34].

Exploring antimicrobial properties along with the antioxidant activity of the plant are an important aspect as there is a growing need to replace existing synthetic food additives with those of natural origin. Various studies have demonstrated the antibacterial potential of different species of *Capsicum* spp. Methanolic extracts of *C. annuum* and *C. frutescens* were found effective against food-borne pathogens *Staphylococcus aureus*, *Vibrio cholerae*, and Salmonella Typhimurium. Recently, an aqueous extract of yellow-colored *C. annuum* was found to demonstrate the highest antimicrobial activity against pathogen *P. aeruginosa*. In another study, phenolic compounds capsaicin, dihydrocapsaicin, and chrysoeriol isolated from the hexane and acetonitrile extracts of fruit, peel, and seed of *C. frutescens* demonstrated promising antimicrobial activity against three Gram-negative bacteria (*E. coli*, *P. aeruginosa,* and *K. pneumoniae*), three Gram-positive bacteria (*Enterococcus faecalis*, *Bacillus subtilis,* and *S. aureus*), and yeast (*C. albicans*). Flavanoid chrysoeriol was found to possess potent antimicrobial potential as compared to the other two isolated compounds.

This study was undertaken to investigate the effect of different solvents extractions (methanol, ethanol, and water) on the antioxidant and antimicrobial properties of oven-dried red, yellow, and green peppers.

## 2. Materials and Methods

### 2.1. Plant Materials and Reagents

Fresh red, yellow, and green peppers were procured from the local market in Riyadh, Saudi Arabia, in January 2021. The experiments were performed immediately after procurement. Gallic acid, Folin–Ciocalteu reagent, and 2,2-diphenyl-1-picrylhydrazyl (DPPH) were purchased from Sigma Chemical Co. (St. Louis, MO, USA).

### 2.2. Sample Preparation

The peppers were rinsed in water and dried on paper towels. The stem and seeds were removed, and edible parts were collected. These portions were cut in almost equally shaped small pieces (2 × 2 cm) and a lot was dried through a hot air oven. All experiments were performed in triplicate, each using 200 g of pepper.

### 2.3. Drying

Two hundred grams of sliced peppers were placed in a hot air oven and dried at 60°C for 4 consecutive days. The dried peppers were allowed to cool down at room temperature for 15 min, and then slices were ground using an electronic blender to obtain peppers powder. Finally, the powder sample was packed in low-density polyethylene (LDPE) bags.

### 2.4. Extraction

Two grams of dried pepper samples were extracted individually with 100 mL of ethanol, methanol, and water solvents. The contents were sonicated at room temperature for 30 min in an ultrasonic bath (frequency, 40 Hz; power, 300 W; SD-350H; Seong Dong, Seoul, Korea) and then filtered using Whatman No. 4 filter paper.

### 2.5. Total Polyphenol Content (TPC)

In this study, TPC was detected by Folin–Ciocalteu (FC) method as described earlier [35]. Firstly, 25 *μ*L of the extract was added to 125 *μ*L of undiluted FC reagent, and then 1500 *μ*L nanopure water was added to the mixture. The mixture was allowed to shake for 1 min at room temperature and then 20% sodium carbonate (375 *μ*L) and 475 *μ*L of water were added, and the final volume of the mixture was made to 2500 *μ*L. Finally, the prepared mixture was incubated at room temperature for 30 min. Phenol's detection was accomplished spectroscopically at 760 nm (Jasco, V-630 spectrophotometer, USA). The TPC was expressed as gallic acid equivalent per Gram dry weight of the sample (mg GAE/g DW).

### 2.6. Total Flavonoid Content (TFC)

The TFC was determined according to the precisely described method used by [35]. Thousand *μ*L of water was added to 250 *μ*L of extract. After that, 75 *μ*L of each 5% (w/v) sodium nitrite and 10% (w/v) aluminum chloride was added to the mixture and incubated for 5 min at room temperature. Then, the mixture was vortexed after adding 500 *μ*L of 1 M sodium hydroxide and 600 *μ*L of water. The blank was prepared following the same procedure without extract. The absorbance was measured spectroscopically at 510 nm (Jasco, V-630 spectrophotometer, USA). TFC was expressed as mg catechin equivalent per Gram dry weight of the sample (mg CE/g DW).

### 2.7. DPPH Scavenging

The free radical scavenging capacity of the extract was determined using DPPH according to the standard method with some modifications [36]. Firstly, 0.1 mM DPPH solution was prepared and then 130 *μ*L of the extract was mixed with 2000 *μ*L of DPPH solution. The mixture was allowed to rest in the dark for 30 min and then absorbance was measured at 510 nm (Jasco, V-630 spectrophotometer, USA). Control was prepared in the same manner, but ethanol was used instead of extract. Methanol was used as a blank. The scavenging percentage was calculated as

(1) $$\begin{array}{c} \text{DPPH scavenging }\%=\frac{\text{Acontrol}-\text{Asample}}{\text{Acontrol}}\times 100. \end{array}$$

#### 2.7.1. Reducing Power

The ferric reducing power of the sample was estimated according to the method of Hayat et al. [33]. Half (0.5) mL extract was mixed with 1.25 mL of potassium ferricyanide, and 1.25 mL buffer (0.2 M, pH 6.6). The mixture was then incubated for 20 min at 50°C. After the incubation of 20 minutes, trichloroacetic acid (1.25 mL) was added and the mixture was centrifuged at 3000 ×g for 10 min at room temperature. Finally, an aliquot (1.25 mL) was taken from the supernatant, to which 1.25 mL water and 0.25 mL of ferric chloride were added. Blank was also prepared following the same protocol but without extract. The absorbance was recorded at 700 nm (Jasco, V-630 spectrophotometer, USA).

### 2.8. Determination of Phenolic Compounds

In the present study, we utilize HPLC with the method described previously [37]. Phenolic compounds (tannic acid, resorcinol, 1,2-dihydroxybenzene, chlorogenic acid, caffeic acid, vanillin, acetylsalicylic acid, 3,5-dinitro salicylic acid, salicylic acid, and quercetin) quantification in three pepper (green, yellow, and red) samples was carried out using HPLC analysis, as described earlier with some modification [37]. The HPLC (Prominence) system Shimadzu (Kyoto, Japan) was equipped with an LC-20AB binary pump and variable Shimadzu SPD-10A UV detector. The column used was Zorbax SB-C18 (250 × 4.6 mm, 5 *μ*m; Agilent, Santa Clara, CA, USA) and the mobile-phase was Milli Q water (1% acetic acid, A) and methanol (B). The binary gradient program used was 0–10 min, 15–30% B; 10–20 min, 30–40% B; 20–30 min, 40–50% B; 30–41 min, 50–60% B; and 41–45 min, 15% B. The flow rate was 1.0 mL/min. The injection volume was 10 *μ*L, and the detector was set at 280 nm. Compounds in pepper samples were identified by comparing their peak retention time with those of standards. All samples were analyzed in duplicate and arithmetical mean ± standard error was reported.

### 2.9. Antimicrobial Activity of Pepper Extracts

Antimicrobial activity of red, green, and yellow pepper extracts was assessed against *Staphylococcus aureus, Listeria monocytogenes, Pseudomonas aeruginosa, Escherichia coli,* and *Candida albicans* using agar well diffusion assay [38]. Briefly, 0.1 ml of overnight grown cultures was spread onto Mueller Hinton Agar (MHA) plates, agar wells were punched, and 6 mg/ml concentration of the prepared extracts was loaded in each well. Solvent (5% DMSO) and Mueller Hinton Broth (MHB) were used as negative controls and antibiotics were used as a positive control. Plates were incubated for 18–24 h at 37°C and observed for halo zones of inhibition around the well. All the samples were analyzed in triplicates.

### 2.10. Assessment of Minimum Inhibitory Concentration (MIC)

MIC of the pepper extracts was determined using the microbroth dilution method described previously [39].

### 2.11. Statistical Analysis

Statistical analysis was performed using SAS (Version 9.2, 2000–2008; SAS Institute Inc., Cary, NC, USA) for data analysis. All the analyses were carried out in triplicate. The results were expressed as mean ± standard deviation (SD). The differences among the treatment groups were analyzed using one-way analysis of variance (ANOVA) at a significance level of *p* ≤ 0.05, and a post hoc analysis using Duncan's multiple range tests was performed if differences were found significant between the groups.

## 3. Results and Discussion

### 3.1. Total Polyphenol Content (TPC)

**Figure 1:**
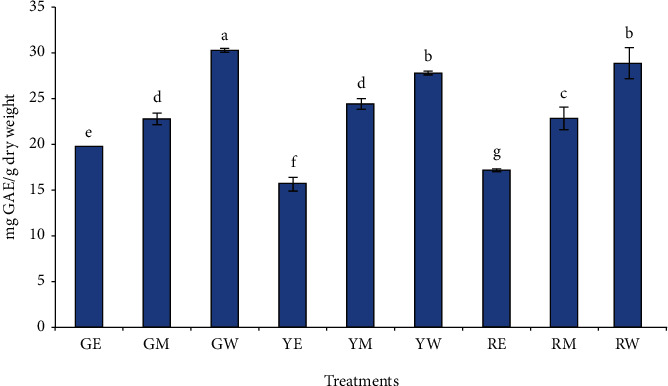
Effect of different solvent extractions on the total polyphenol content of peppers. The treatment codes denoted by two letters represent the green (G), yellow (Y), and red pepper (R), extracted with ethanol (E), methanol (M), and water (W).

The effect of different extraction (ethanol, methanol, and water) solvents on the total polyphenol content of green, yellow, and red peppers are shown in Figure 1. The green pepper water extract showed the highest total polyphenol content (30.15 mg GAE/g DW) followed by red pepper water extract (28.73 mg GAE/g DW) and yellow pepper water extract (27.68 mg GAE/g DW), respectively. The methanol extracts of all the pepper samples showed higher TPC as compared to the ethanol extract. For example, the TPC of methanol extract of green, yellow, and red pepper was 22.69, 24.33, and 22.76, while that of the ethanol extract was 19.63, 15.55, and 17.1 mg GAE/g DW. Our results are contrary to the findings of Sun et al. [14] who reported a higher TPC of red peppers than the green peppers. The TPC of the methanolic extract of green, yellow, orange, and red peppers was documented as 2.4, 3.3, 3.4, and 4.2 micromol catechin equivalent/g fresh weight, respectively. Another study also reported that the methanolic extract of red sweet pepper cultivar/rootstock (Fascinato/Robusto) had a higher concentration of total phenols of 111.26 mg/100 g of dry weight as compared to the green pepper (Sweet/Robusto) which showed the lowest content, averaging 70.39 mg/100 g of dry weight. Moreover, the total phenol content depended on the variety as well as the color of the bell peppers and the highest content was recorded in colored peppers than in the green, values being highest in red, followed by yellow, and then by orange peppers [18]. But our results are in line with the findings of Blanco-Ríos et al. [40], who found that the variety Orion (green) had the highest concentration of phenolic compounds, while no differences were detected between the varieties Mazurca (red), Simpaty (orange), and Taranto (yellow). Ahmad et al. [41] reported that the solvents (acetone, ethanol, and water) established a significant role in the extraction of phenolic compounds from 27 samples of pepper from different origins. Kumar et al. [42] reported that the fresh green bell peppers showed a TPC of 64.58 mg GAE/g. The extraction for the TPC measurement in this study was performed by homogenizing the fresh bell peppers with water.

### 3.2. Total Flavonoid Content (TFC)

**Figure 2:**
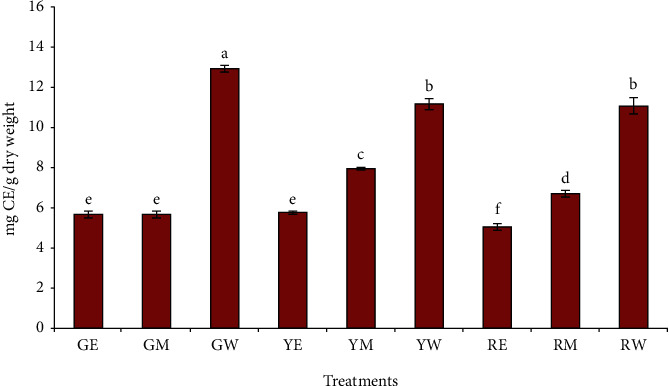
Effect of different solvent extractions on the total flavonoid content of peppers. The treatment codes denoted by two letters represent the green (G), yellow (Y), and red pepper (R), extracted with ethanol (E), methanol (M), and water (W).

Figure 2 shows the total flavonoid content of pepper samples extracted with three different solvents. TFC showed almost a similar trend as that of the TPC. The water extract of green pepper exhibited the highest (13.04 mg CE/g DW), while the ethanol extract of red pepper showed the lowest (5.11 mg CE/g DW) total flavonoid content among all the samples. Statistically, the total flavonoid contents of the methanol extract of green pepper (5.74 mg CE/g DW) and ethanol extracts of green (5.72 mg CE/g DW) and yellow pepper (5.82 mg CE/g DW) were not significantly different from each other (*p* > 0.05), while the TPC of the water extracts of yellow and red peppers were also statistically similar to each other. Kumar et al. [41] reported the TFC of water extract of green bell-pepper as 11.95 mg quercetin equivalent (QE)/g sample. In an earlier study, the TFC of the water, methanol, and ethanol extract of the pepper (*C. annum* L.) was determined as 78.2, 67.2, and 82.3 mg QE/100 g DW, respectively, and the values were not significantly different (*p* > 0.05) from each other [43]. Previous study reported that the TFC values of extract from *Capsicum annuum* L. averaged from 121 to 130 mg QE/100 DW [44]. It is well known that water is more polar than ethanol and methanol. Some of the plant bioactive compounds, like O-methylated are less polar than the nonmethylated flavonoids [45]. Based on the different TFC valued obtained by solvent used, the results of our study might be explained that the peppers have different group of flavonoids soluble in different polarities. Moreover, the different values of bioactive compounds of pepper reported in the literature might be due to the varietal, agronomical, environmental, and analytical factor. Hallman and Rembialkowska [46] reported that the phenolic content was influenced by the crop, as the organic system gave higher values than did the conventional one.

### 3.3. Antioxidant Activity

**Figure 3:**
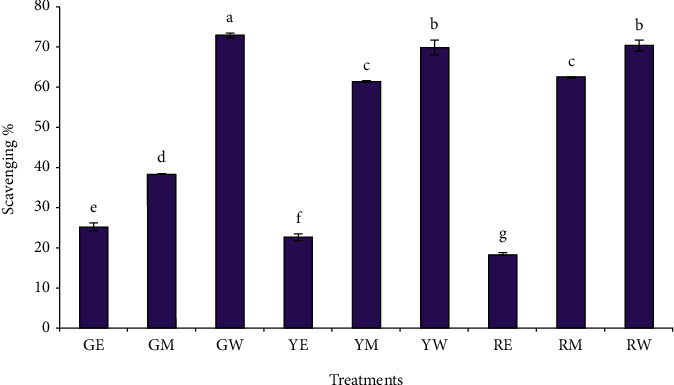
Effect of different solvent extractions on the DPPH scavenging activity of peppers. The treatment codes denoted by two letters represent the green (G), yellow (Y), and red pepper (R), extracted with ethanol (E), methanol (M), and water (W).

**Figure 4:**
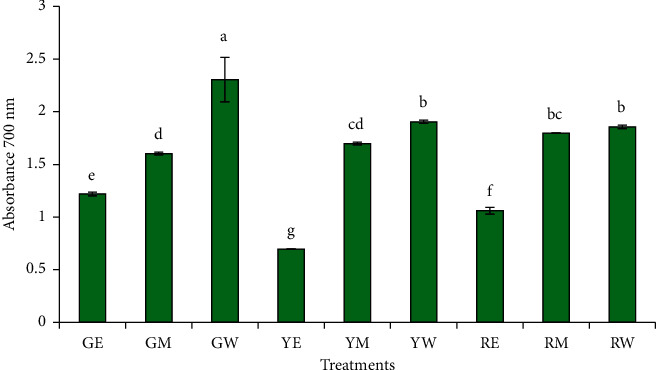
Effect of different solvent extractions on the reducing power of peppers. The treatment codes denoted by two letters represent the green (G), yellow (Y), and red pepper (R), extracted with ethanol (E), methanol (M), and water (W).

The antioxidant potential of different extracts of green, yellow, and red peppers are as assessed by 2,2-diphenyl-1-picrylhydrazyl scavenging and ferric reducing power is shown in Figure 3, respectively. The antioxidant activity potential of the extracts echoed the aforementioned results of TPC and TFC. The significantly highest DPPH scavenging was shown by the water extract (0.02 g/mL) of green pepper (72.76%) (*p* < 0.05), which was followed by the water extract of red pepper (70.26%) and yellow pepper, respectively. But statistically, there was no difference (*p* > 0.05) between the DPPH scavenging of the water extract of the red and yellow pepper. The ethanol extract of red pepper showed the lowest DPPH scavenging (18.31%) among all the samples.  Figure 4 depicts the reducing power of the pepper extracts. As can be seen, the highest reducing power was exhibited by the water extract of green pepper (2.305) followed by the water extract of yellow pepper (1.905) and red pepper (1.857), respectively. The lowest reducing power was shown by the ethanol extract of yellow pepper (0.696) among all the samples. The higher antioxidant activity of the water extract could be due to the leaching of hydrophilic phenolic compounds in the extract [47, 48].

A recent study reported the DPPH scavenging of 88.35% for the water extract (0.25 g/mL) of green bell pepper [42]. In another study, the methanolic extract (0.04 g/mL) of red bell pepper dried at 50°C and 70°C exhibited the DPPH scavenging of 67.02% and 73.25%, respectively [49]. The free radical scavenging ability of peppers determined by the DPPH method was the lowest for the green pepper but not significantly different from the other 3 peppers (yellow, orange, and red) (Sun et al.) [14]. In another study, the TPC, TFC, and DPPH scavenging of red and green sweet bell peppers processed at various temperatures were evaluated. The methanolic extract of red peppers showed higher DPPH scavenging under all the processing conditions as compared to the green peppers (Yazdizadeh Shotorbani et al.) [50]. Chávez-Mendoza et al. [18] evaluated the antioxidant activity by DPPH of the 80% ethanolic extract of different cultivar/rootstock combinations of bell peppers and found that Fascinato/Robusto red colored had the highest antioxidant activity with an average of 79.65%, while yellow colored Jeanette/Terrano presented the lowest activity of 64.90%. The average antioxidant activity of the cultivar/rootstock combinations is diminished as follows: (red)Fascinato/Robusto > (red)Fascinato/Terrano > (green)Sweet/Robusto > (orange)Orangela/Terrano > (yellow)Jeanette/Terrano.

### 3.4. Reducing Power

#### 3.4.1. Antimicrobial Studies

**Figure 5:**
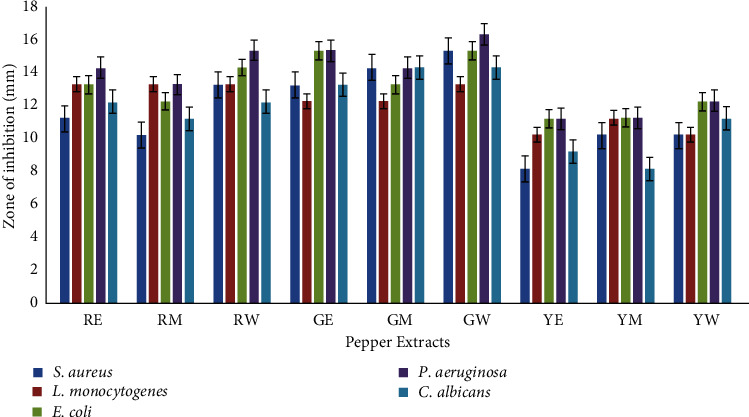
Antimicrobial activity of pepper extracts. The treatment codes denoted by two letters represent the green (G), yellow (Y), and red pepper (R), extracted with ethanol (E), methanol (M), and water (W).

Solvent extracts of red, yellow, and green pepper were examined for their potential antimicrobial activity against bacterial and fungal pathogens. Aqueous extracts of all the three pepper samples exhibited slightly higher inhibition zones as compared to their corresponding ethanolic and methanolic extract (Figure 5). Aqueous extract of green pepper extract demonstrated the highest inhibition zone of 15, 13, 15, 14, and 12 mm against *S. aureus, L. monocytogenes*, *E. coli, P. aeruginosa,* and *C. albicans*, respectively. Similarly, the zone of inhibition for green pepper (ethanol extract) was recorded as 13, 12, 15, 15, and 13 mm against *S. aureus, L. monocytogenes, E. coli, P. aeruginosa,* and *C. albicans*, respectively, while methanolic extract of green pepper demonstrated inhibition zone ranging from 10 to 13 mm against the test pathogens. Red pepper (alcoholic extracts) showed inhibition zones ranging from 10 to 14 mm, while the aqueous extract of the red pepper demonstrated inhibition zones of 12–15 mm against the test pathogens. In the case of yellow pepper, extract from methanolic samples showed the highest zone of 11 mm against *E. coli*, *L. monocytogenes*, and *P. aeruginosa,* and the lowest zone of 8 mm was recorded against *C. albicans*. Almost similar results were observed with the ethanolic extract of yellow pepper. Slightly higher inhibition zones ranging from 10 to 12 mm were recorded with the aqueous extract of yellow pepper samples. Antibiotics chloramphenicol and fluconazole (antifungal) were used as positive controls. Our findings are in sync with those reported with methanolic extracts of *C. annuum* and *C. frutescens*. Both extracts were found effective against food-borne pathogens *Staphylococcus aureus, Vibrio cholerae,* and *Salmonella typhimurium* [51]. Recently, an aqueous extract of yellow-colored *C. annuum* was found to demonstrate the highest antimicrobial activity against pathogen *P. aeruginosa* [52]. In another study, phenolic compounds capsaicin, dihydrocapsaicin, and chrysoeriol isolated from the hexane and acetonitrile extracts of fruit, peel, and seed of *C. frutescens* demonstrated promising antimicrobial activity against three Gram-negative bacteria (*E. coli*, *P. aeruginosa,* and *K. pneumoniae*), three Gram-positive bacteria (*Enterococcus faecalis*, *Bacillus subtilis,* and *S. aureus*), and yeast (*C. albicans*) [53].

MIC and MBC values of all the prepared extracts were determined against all test pathogens. Extracts of water demonstrated lower MIC and MBC values as compared to the alcoholic extracts (Table 1). The lowest MIC values ranging from 0.5 to 2.0 mg/ml concentration were recorded for aqueous extracts of green pepper, while the highest MICs (4–8 mg/ml) and MBCs (8–16 mg/ml) were observed with the alcoholic extracts of yellow pepper. The antimicrobial action of the pepper extracts can be attributed to the presence of polyphenols and capsaicinoids as demonstrated previously by Mokhtar et al. [54]. Our results demonstrate slightly higher MIC values against Gram-positive bacteria as compared to Gram-negative bacteria. This finding is on the expected lines as the structure and composition of the cell wall of Gram-positive bacteria differs from Gram-negative bacteria. The cell wall of the Gram-positive bacteria comprises a thick layer of peptidoglycan with covalently bound teichuronic and teichoic acid making them less susceptible to the action of plant extracts.

### 3.5. HPLC Analysis of Phenolic Compounds

**Figure 6:**
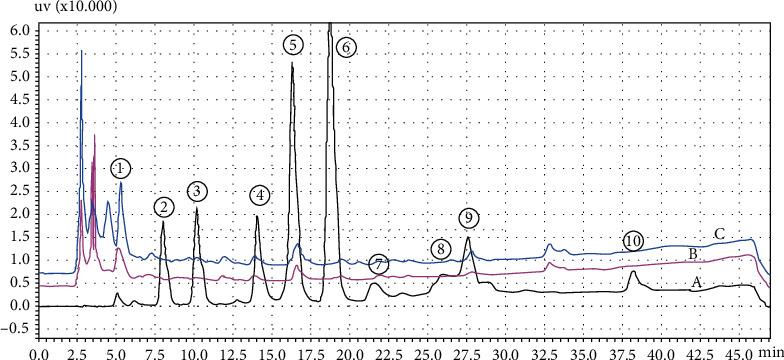
Representative HPLC chromatogram of phenolic compound analysis. 1 = tannic acid, 2 = resorcinol, 3 = 1,2-DHB, 4 = chlorogenic acid, 5 = caffeic acid, 6 = vanillin, 7 = acetyl salicylic acid, 8 = 3,5-DNSA, 9 = salicylic acid, 10 = quercetin. A = phenolic compound standards (50 *μ*g/ml), B = green pepper ethanol extraction, and C = green pepper methanol extraction.

The effect of different extracting solvents on the phenolic compounds of three (green, yellow, and red) pepper (*Capsicum annuum* L.) samples that were analyzed by high-performance liquid chromatography (HPLC) representative overlay chromatograms is shown in Figure 6 and the average values are reported in Table 2. Phenolic substances' type and concentration are responsible for biological activities. The analysis and characterization of phenolic compounds with modern techniques potentially open the door for the discovery of biologically active compounds. The factors which affect the phenolic compounds are the production system, climate conditions, fruits, cultivars' maturation state, and postharvest treatment [17]. Tannic acid is the major phenolic compound in all three pepper samples ranging within 1028.67–3501.16 mg/100 g dw. Chlorogenic acid, 19.03–28.42 mg/100 g dw, is high in green pepper as compared to yellow and red pepper samples. Other individual phenolic compound ranges are resorcinol 10.42–14.45, 1,2-DHB 10.57–21.47, caffeic acid 16.45–30.23, acetylsalicylic acid 9.94–34.94, 3, 5 DNSA 4.10–18.51, and salicylic acid 0.5–27.83 mg/100 g dw. In general, yellow pepper samples show higher phenolic compounds as compared to red and green peppers. Green pepper phenolic compounds are higher in water extraction than methanol and ethanol extraction. Guilherme et al. [17] reported the higher content of chlorogenic acid in green pepper. Hallmann and Rembialkowska [46] reported chlorogenic acid 877.0 mg/kg dw in organic and 749.0 mg/kg in conventional grown bell pepper. Lee et al. [55] reported that the fresh pepper contains the total soluble phenolic compound from 178 to 384.9 mg chlorogenic acid equivalent per 100 gram off fresh weight. Caffeic acid in four cultivars of red sweet pepper are a little higher to this study, in the range of 38–63 *μ*g/kg [56]. Different values in the literature may be due to different cultivars, different extraction methods, and the ways to express the results [57].

## 4. Conclusions

The current study investigated the effect of different solvents extractions (methanol, ethanol, and water) and oven drying on the antioxidant and antimicrobial properties of red, yellow, and green peppers. All solvent extracts impacted the biological properties of the pepper samples. Among all the samples tested, an aqueous extract of green pepper was found to possess the highest TPC, TFC, antioxidant, and antimicrobial activity. HPLC analysis revealed tannic acid as the major phenolic compound in all three pepper samples, while chlorogenic acid was found to be in higher amounts in green pepper samples as compared to red and yellow pepper. It is postulated that envisaged that the microwave drying/heating technique can improve the antioxidant and antimicrobial activity of the pepper, but the exact mechanism needs to be unearthed in future studies. Further, the findings of this study could be exploited in the processing, storage, and consumption of pepper.

## Figures and Tables

**Table 1:** MIC and MBC values of different pepper extracts against test pathogens.

Sample	Pathogens									
	*S. aureus*		*L. monocytogenes*		*E. coli*		*P. aeruginosa*		*C. albicans*	
	MIC	MBC	MIC	MBC	MIC	MBC	MIC	MBC	MIC	MBC
RE	4	8	4	8	1	2	2	4	8	16
RM	4	8	4	8	1	2	2	4	8	16
RW	2	8	2	4	1	2	1	2	2	4
GE	2	4	2	4	1	2	2	4	4	8
GM	2	4	2	4	1	2	2	4	4	4
GW	1	2	1	1	0.5	1	0.5	1	2	2
YE	8	16	8	16	4	8	4	4	8	16
YM	8	8	8	16	4	8	4	4	8	16
YW	4	8	4	8	2	4	2	4	4	8

MIC and MBC values are given in mg/ml.

**Table 2:** Phenolic compounds of pepper (green, yellow, and red) HPLC (mg/100 g) dry weight (dw).

Sample	Tannic acid	Resorcinol	1,2-DHB	Chlorogenic acid	Caffeic acid	Vanillin	Acetyl salicylic acid	3,5-DNSA	Salicylic acid
GE	1028.67 ± 1.38	ND	10.57 ± 0.10	19.03 ± 0.28	16.45 ± 0.19	1.43 ± 0.02	34.94 ± 0.76	5.72 ± 0.08	13.53 ± 0.15
GM	1689.40 ± 1.37	ND	14.34 ± 0.11	25.42 ± 0.51	24.20 ± 0.32	2.44 ± 0.03	22.77 ± 0.25	9.15 ± 0.14	27.83 ± 0.38
GW	2284.25 ± 1.84	ND	21.47 ± 0.09	28.42 ± 0.13	23.05 ± 0.10	2.42 ± 0.00	28.07 ± 0.03	9.92 ± 0.07	17.83 ± 0.22
YE	2577.62 ± 1.57	ND	13.88 ± 0.01	10.44 ± 0.01	18.89 ± 0.04	0.70 ± 0.04	11.88 ± 0.37	4.10 ± 0.03	2.78 ± 0.02
YM	3501.16 ± 1.23	ND	13.31 ± 0.00	13.81 ± 0.10	28.79 ± 0.10	2.01 ± 0.08	ND	17.06 ± 0.01	5.07 ± 0.02
YW	2618.90 ± 3.54	14.45 ± 0.18	18.40 ± 0.04	15.03 ± 0.02	26.70 ± 0.03	1.64 ± 0.01	ND	18.31 ± 0.11	2.96 ± 0.01
RE	2559.68 ± 1.19	ND	12.67 ± 0.00	7.99 ± 0.08	21.36 ± 0.06	0.68 ± 0.02	9.94 ± 0.04	6.10 ± 0.02	1.06 ± 0.00
RM	2940.58 ± 1.05	ND	17.92 ± 0.07	13.14 ± 0.00	30.23 ± 0.03	0.55 ± 0.01	ND	18.51 ± 0.09	0.85 ± 0.00
RW	1933.00 ± 3.57	10.42 ± 0.16	21.15 ± 0.26	16.15 ± 0.09	27.47 ± 0.07	1.61 ± 0.09	ND	15.01 ± 0.02	0.50 ± 0.00

DHB = dihydroxy benzene; DNSA = dinitro salicylic acid; G = green pepper; Y = yellow pepper; R = red pepper; E = ethanol; M = methanol; W = water; ND = not detected.

## Data Availability

Data used to support the findings are included within the article.

## References

[1] Wong P. Y., Tan S. T. (2020). Comparison of total phenolic content and antioxidant activities in selected coloured plants. *British Food Journal*.

[2] Gülçin İ. (2012). Antioxidant activity of food constituents: an overview. *Archives of Toxicology*.

[3] Unuofin J. O., Otunola G. A., Afolayan A. J. (2017). Phytochemical screening and in vitro evaluation of antioxidant and antimicrobial activities of Kedrostis africana (L.) Cogn. *Asian Pacific Journal of Tropical Biomedicine*.

[4] Pennington J. A. T., Fisher R. A. (2009). Classification of fruits and vegetables. *Journal of Food Composition and Analysis*.

[5] Hurtado-Barroso S., Trius-Soler M., Lamuela-Raventós R. M., Zamora-Ros R. (2020). Vegetable and fruit consumption and prognosis among cancer survivors: a systematic review and meta-analysis of cohort studies. *Advances in Nutrition*.

[6] Zurbau A., Au‐Yeung F., Blanco Mejia S. (Oct. 2020). Relation of different fruit and vegetable sources with incident cardiovascular outcomes: a systematic review and meta‐analysis of prospective cohort studies. *Journal of American Heart Association*.

[7] Igbokwe G., Anagonye C. (2013). Determination of *β*–carotene & vitamin C content of fresh green pepper (capsicum annnum), fresh red pepper (capsicum annum) and fresh tomatoes (solanumly copersicum) fruits. *Bioscience Journal*.

[8] Nazzaro F., Caliendo G., Arnesi G., Veronesi A., Sarzi P., Fratianni F. (2009). Comparative content of some bioactive compounds in two varieties of *Capsicum annuum* L. Sweet pepper and evaluation of their antimicrobial and mutagenic activities. *Journal of Food Biochemistry*.

[9] Marín A., Ferreres F., Tomás-Barberán F. A., Gil M. I. (2004). Characterization and quantitation of antioxidant constituents of sweet pepper (Capsicum annuum L.). *Journal of Agricultural and Food Chemistry*.

[10] Howard L., Wildman R. (2001). Antioxidant vitamin and phytochemical content of fresh and processed pepper fruit (Capsicum annuum). *Handbook of Nutraceuticals and Functional Foods*.

[11] Mohd Hassan N., Yusof N. A., Yahaya A. F., Mohd Rozali N. N., Othman R. (Oct. 2019). Carotenoids of capsicum fruits: pigment profile and health-promoting functional attributes. *Antioxidants*.

[12] Howard L., Wildman R., Wildman R. (2006). Antioxidant vitamin and phytochemical content of fresh and processed pepper fruit (Capsicum annuum). *Handbook of Nutraceuticals and Functional Foods*.

[13] Matsufuji H., Ishikawa K., Nunomura O., Chino M., Takeda M. (2007). Anti-oxidant content of different coloured sweet peppers, white, green, yellow, orange and red (*Capsicum annuum* L.). *International Journal of Food Science and Technology*.

[14] Sun T., Xu Z., Wu C.-T. (2007). Antioxidant activities of different colored sweet bell peppers (*Capsicum annuum* L.). *Journal of Food Science*.

[15] Materska M., Perucka I. (2005). Antioxidant activity of the main phenolic compounds isolated from hot pepper fruit (capsicum annuum L.). *Journal of Agricultural and Food Chemistry*.

[16] Hasler C. (1998). Functional foods: their role in disease prevention and health promotion. *Food Technology*.

[17] Guilherme R., Aires A., Rodrigues N., Peres A. M., Pereira J. A. (2020). Phenolics and antioxidant activity of green and red sweet peppers from organic and conventional agriculture: a comparative study. *Agriculture*.

[18] Chávez-Mendoza C., Sanchez E., Muñoz-Marquez E., Sida-Arreola J., Flores-Cordova M. (2015). Bioactive compounds and antioxidant activity in different grafted varieties of bell pepper. *Antioxidants*.

[19] Parisi M., Alioto D., Tripodi P. (2020). Overview of biotic stresses in pepper (capsicum spp.): sources of genetic resistance, molecular breeding and genomics. *International Journal of Molecular Sciences*.

[20] Byun E. B., Park W. Y., Kim W. S., Song H. Y., Sung N. Y., Byun E. H. (2018). Neuroprotective effect of Capsicum annuum var. abbreviatum against hydrogen peroxide-induced oxidative stress in HT22 hippocampus cells. *Bioscience, Biotechnology, and Biochemistry*.

[21] Nagasukeerthi P., Mooventhan A., Manjunath N. K. (2017). Short-term effect of add on bell pepper (Capsicum annuum var. grossum) juice with integrated approach of yoga therapy on blood glucose levels and cardiovascular functions in patients with type 2 diabetes mellitus: a randomized controlled study. *Complementary Therapies in Medicine*.

[22] Bosland P., Janick J. (1996). Capsicums: innovative uses of an ancient crop. *Progress in New Crops*.

[23] Loizzo M. R., Tundis R., Menichini F., Statti G. A., Menichini F. (2008). Influence of ripening stage on health benefits properties of capsicum annuum var. Acuminatum L.: in vitro studies. *Journal of Medicinal Food*.

[24] Srinivasan K. (2005). Spices as influencers of body metabolism: an overview of three decades of research. *Food Research International*.

[25] Aizawa K., Inakuma T. (2009). Dietary capsanthin, the main carotenoid in paprika (Capsicum annuum), alters plasma high-density lipoprotein-cholesterol levels and hepatic gene expression in rats. *British Journal of Nutrition*.

[26] Oboh G., Rocha J. B. T. (2008). Hot pepper (capsicum spp.) protects brain from sodium nitroprusside- and quinolinic acid-induced oxidative stress in vitro. *Journal of Medicinal Food*.

[27] Bettaieb Rebey I., Bourgou S., Ben Slimen Debez I. (2012). Effects of extraction solvents and provenances on phenolic contents and antioxidant activities of cumin (cuminum cyminum L.) seeds. *Food and Bioprocess Technology*.

[28] Chung H., Ji X., Canning C., Sun S., Zhou K. (2010). Comparison of different strategies for soybean antioxidant extraction. *Journal of Agricultural and Food Chemistry*.

[29] Menichini F., Tundis R., Bonesi M. (2009). The influence of fruit ripening on the phytochemical content and biological activity of Capsicum chinense Jacq. cv Habanero. *Food Chemistry*.

[30] Bae H., Jayaprakasha G. K., Crosby K., Jifon J. L., Patil B. S. (2012). Influence of extraction solvents on antioxidant activity and the content of bioactive compounds in non-pungent peppers. *Plant Foods for Human Nutrition*.

[31] Thuphairo K., Sornchan P., Suttisansanee U. (2019). Bioactive compounds, antioxidant activity and inhibition of key enzymes relevant to alzheimer’s disease from sweet pepper (capsicum annuum) extracts. *Preventive Nutrition and Food Science*.

[32] Haghi A. K., Amanifard N. (2008). Analysis of heat and mass transfer during microwave drying of food products. *Brazilian Journal of Chemical Engineering*.

[33] Hayat K., Zhang X., Chen H., Xia S., Jia C., Zhong F. (2010). Liberation and separation of phenolic compounds from citrus Mandarin peels by microwave heating and its effect on antioxidant activity. *Separation and Purification Technology*.

[34] Hayat K., Abbas S., Hussain S., Shahzad S. A., Tahir M. U. (2019). Effect of microwave and conventional oven heating on phenolic constituents, fatty acids, minerals and antioxidant potential of fennel seed. *Industrial Crops and Products*.

[35] Hayat K. (2020). Impact of drying methods on the functional properties of peppermint (mentha piperita L.) leaves. *Science Letter*.

[36] Gulcin I., Kirecci E., Akkemik E., Topal F., Hisar O. (2010). Antioxidant, antibacterial, and anticandidal activities of an aquatic plant: duckweed (Lemna minor L. Lemnaceae). *Turkish Journal of Biology*.

[37] He J., Yin T., Chen Y. (2015). Phenolic compounds and antioxidant activities of edible flowers of Pyrus pashia. *Journal of Functional Foods*.

[38] Ahmad I., Beg A. Z. (2001). Antimicrobial and phytochemical studies on 45 Indian medicinal plants against multi-drug resistant human pathogens. *Journal of Ethnopharmacology*.

[39] Husain F. M., Ahmad I., Khan F. I. (2018). Seed extract of Psoralea corylifolia and its constituent bakuchiol impairs AHL-based quorum sensing and biofilm formation in food- and human-related pathogens. *Frontiers in cellular and infection microbiology*.

[40] Blanco-Ríos A. K., Medina-Juárez L. Á., González-Aguilar G. A., Gámez-Meza N. (2017). Antioxidant activity of the phenolic and oily fractions of different sweet bell peppers. *Journal of the Mexican Chemical Society*.

[41] Ahmad R., Ahmad N., Alkhars S. (2021). Green accelerated solvent extraction (ASE) with solvent and temperature effect and green UHPLC-DAD analysis of phenolics in pepper fruit (Capsicum annum L.). *Journal of Food Composition and Analysis*.

[42] Kumar N., Pratibha N., Ojha A., Upadhyay A., Singh R., Kumar S. (2021). Effect of active chitosan-pullulan composite edible coating enrich with pomegranate peel extract on the storage quality of green bell pepper. *LWT*.

[43] Askin B., Yazici S. O., Yazici S. O. (2021). Bioactive compounds, antioxidant potential and color properties of dried red pepper (capsicum annuum L.). *International Journal of Innovative Approaches in Agricultural Research*.

[44] Zaki N., Hakmaoui A., Ouatmane A., Hasib A., Fernández-Trujillo J. P. (2013). Bioactive components and antioxidant activity of Moroccan Paprika (Capsicum annuum L.) at different period of harvesting and processing. *Journal of Biology, Agriculture and Healthcare*.

[45] Sopee M. S. M., Azlan A., Khoo H. E. (2019). Comparison of antioxidants content and activity of Nephelium mutabile rind extracted using ethanol and water. *Journal of Food Measurement and Characterization*.

[46] Hallmann E., Rembiałkowska E. (2012). Characterisation of antioxidant compounds in sweet bell pepper (Capsicum annuumL.) under organic and conventional growing systems. *Journal of the Science of Food and Agriculture*.

[47] Wang J., Yang X.-H., Mujumdar A. S. (2017). Effects of various blanching methods on weight loss, enzymes inactivation, phytochemical contents, antioxidant capacity, ultrastructure and drying kinetics of red bell pepper (Capsicum annuum L.). *LWT*.

[48] Ornelas-Paz J. d. J., Cira-Chávez L. A., Gardea-Béjar A. A. (2013). Effect of heat treatment on the content of some bioactive compounds and free radical-scavenging activity in pungent and non-pungent peppers. *Food Research International*.

[49] Arslan D., Özcan M. M. (2011). Dehydration of red bell-pepper (Capsicum annuum L.): change in drying behavior, colour and antioxidant content. *Food and Bioproducts Processing*.

[50] Yazdizadeh Shotorbani N., Jamei R., Heidari R. (2013). Antioxidant activities of two sweet pepper Capsicum annuum L. varieties phenolic extracts and the effects of thermal treatment. *Avicenna Journal of Phytomedicine*.

[51] Koffi-Nevry R., Kouassi K. C., Nanga Z. Y., Koussémon M., Loukou G. Y. (2012). Antibacterial activity of two bell pepper extracts:capsicum annuum L. and Capsicum frutescens. *International Journal of Food Properties*.

[52] Samrot A. V., Shobana N., Jenna R. (2018). Antibacterial and antioxidant activity of different staged ripened fruit of capsicum annuum and its green synthesized silver nanoparticles. *Bionanoscience*.

[53] Nascimento P., Nascimento T., Ramos N. (2014). Quantification, antioxidant and antimicrobial activity of phenolics isolated from different extracts of capsicum frutescens (pimenta malagueta). *Molecules*.

[54] Mokhtar M., Ginestra G., Youcefi F., Filocamo A., Bisignano C., Riazi A. (2017). Antimicrobial activity of selected polyphenols and capsaicinoids identified in pepper (capsicum annuum L.) and their possible mode of interaction. *Current Microbiology*.

[55] Lee Y., Howard L. R., Villalón B. (1995). Flavonoids and antioxidant activity of fresh pepper (capsicum annuum) cultivars. *Journal of Food Science*.

[56] Rodrigues C., Nicácio A., Jardim I., Visentainer J., Maldaner L. (2019). Determination of phenolic compounds in red sweet pepper (capsicum annuum L.) using a modified QuEChERS method and UHPLC-MS/MS analysis and its relation to antioxidant activity. *Journal of the Brazilian Chemical Society*.

[57] Zhang D., Hamauzu Y. (2003). Phenolic compounds, ascorbic acid, carotenoids and antioxidant properties of green, red and yellow bell peppers. *Journal of Food Agriculture and Environment*.

